# A clinical analysis of intestinal mucosal necrosis and exfoliation induced by superior mesenteric vein thrombosis: A case report

**DOI:** 10.3389/fsurg.2022.988195

**Published:** 2023-01-09

**Authors:** Bin Lin, Yan-ping Zhang, Lin-ying Xue, Ying Ye, Yi Tang, Chang Shun Yang, Jie-wei Luo, Mei-zhu Gao, Zhu-ting Fang

**Affiliations:** ^1^ Fujian Provincial Hospital, Shengli Clinical Medical College of Fujian Medical University, Fuzhou, China; ^2^ Department of Geriatrics, Fujian Provincial Geriatric Hospital, Fuzhou, China; ^3^ Department of Interventional Radiology, Fujian Provincial Hospital, Fuzhou, China; ^4^ Department of Nephrology, Fujian Provincial Hospital, Fuzhou, China

**Keywords:** thrombosis, intestinal mucosal necrosis, intestinal mucosal exfoliation, intestinal obstruction, case report

## Abstract

**Background:**

Superior mesenteric vein (SMV) thrombosis is a rare intestinal ischemic disease. The clinical manifestations of patients differ, and most experience gastrointestinal symptoms.

**Case summary:**

A 45-year-old female patient presented with persistent abdominal pain and abnormal vaginal bleeding for 7 days. A physical examination revealed significant abdominal tenderness with positive rebound tenderness. A laboratory examination revealed a white blood cell count of 27 × 10^9^/l, hemoglobin level of 52 g/L, and D-dimer of 4.54 mg/l. Enhanced computed tomography revealed a thickening and swelling of the jejunum and ileum in the left upper quadrant and portal vein. Filling defects in the main lumen and branch lumen suggested the possibility of portal vein and superior mesenteric vein thrombosis. Symptoms improved after treatment with low-molecular-weight heparin and warfarin. One month later, the patient developed occasional dull pain in the left lower quadrant, with long strips of discharge. An electronic colonoscopy revealed avascular necrosis and tissue exfoliation of the intestinal mucosa. After the continuation of warfarin therapy, the abdominal pain resolved. Five months later, the patient experienced recurrent abdominal pain and vomiting. A physical examination revealed a blood pressure of 75/49 mm Hg. An incomplete ileus with the portal and superior mesenteric vein thrombosis was diagnosed, partial jejunectomy and gastrointestinal bypass anastomosis were performed, and warfarin was continued postoperatively.

**Conclusion:**

The intestinal mucosal shedding observed, in this case, was caused by SMV thrombosis, which enriched the clinical manifestations of the disease and provided a new basis for the clinical diagnosis of SMV thrombosis.

## Introduction

Superior mesenteric vein (SMV) thrombosis is a rare intestinal ischemic disease, and its main clinical manifestations are abdominal pain, nausea, vomiting, and other gastrointestinal symptoms. Due to atypical clinical symptoms leading to an untimely diagnosis and delayed treatment, the mortality rate of SMV thrombosis has increased, accounting for approximately 5%–15% of mesenteric thromboembolic events (1). Patients with hereditary anticoagulation dysfunction, cancer, and postsurgery conditions are at a high risk of SMV thrombosis (2). Some studies have found that infections such as influenza and COVID-19 can also induce SMV thrombosis in some patients (3, 4). Treatment mainly involves heparin for systemic anticoagulation, thrombolysis, and interventional therapy. According to the patient’s hypercoagulable state and risk factors, clinicians should take a decision on whether to place the patient on lifelong oral anticoagulant therapy (4). Patients with SMV thrombosis show characteristics of other diseases such as appendicitis because of the location of the thrombus (5). This study found a patient with recurrent SMV thrombosis that mainly manifested as abdominal pain, large necrosis, and shedding of the intestinal mucosa. The patient signed an informed consent form and this case was approved by the institutional review board.

## Case presentation

**Figure 1:**
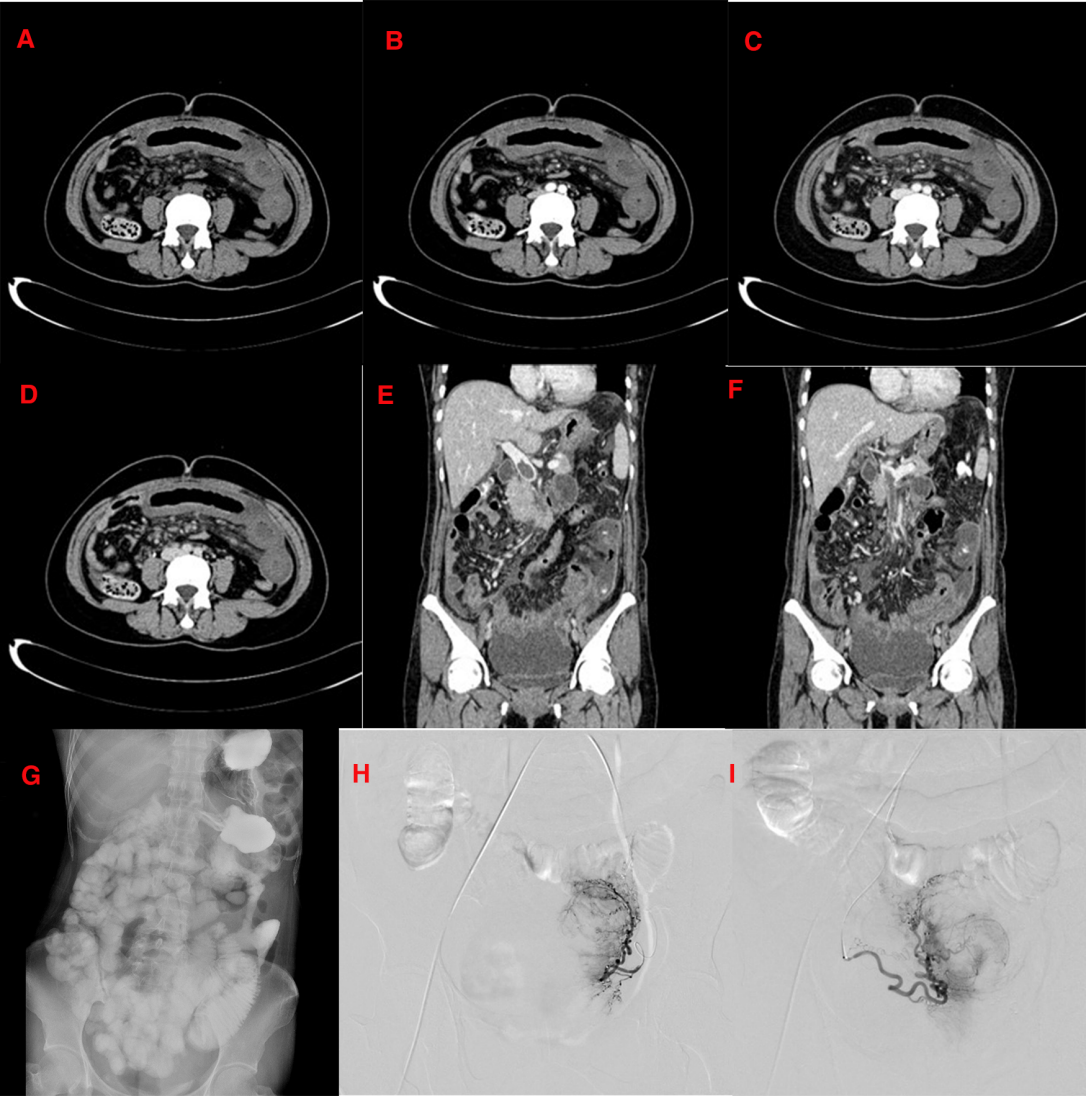
(December 15, 2016) CT scan of the patient on first admission. (**A–F**): The CT shows that the left middle and upper abdomen is partially empty, and the ileal bowel wall is thickened and swollen; filling defects can be seen in the lumen of the main portal vein and its branches and the superior mesenteric vein and its branches. A small amount of effusion is seen around the liver, spleen, and intestine. The enhancement of the right hepatic lobe is not uniform in the arterial phase of the enhanced scan, and the portal venous phase and the delayed phase are in isodensity. A small circular low-density shadow with a diameter of approximately 0.5 cm is seen in the IV segment of the liver near the diaphragm. There is no enhancement on the enhanced scan. The uterine shadow is enlarged, its internal density is uneven, and multiple lumpy and patchy areas of reduced density can be seen. The enhancement scan is uneven; (**G**): The upright abdominal plain film shows that the intestinal tube in the abdomen is slightly dilated. (**H**): Uterine arteriography shows that the left and right uterine arteries are thickened and disordered, and the uterus is enlarged in the parenchymal stage. (**I**): After uterine artery embolization, most left and right uterine arteries are blocked.

A 45-year-old woman presented to the emergency department of our hospital with a chief complaint of persistent abdominal pain and vaginal bleeding for 7 days. A physical examination after admission revealed a marked abdominal tenderness with positive rebound tenderness. Blood tests showed WBC 27 × 109/L, N 87.4%, Hb 52 g/L, and Plt 371 × 109/L. Abdominal color Doppler ultrasound showed ascites, a slightly dilated lower abdominal bowel, and multiple hypoechoic myometria, following which the possibility of the presence of fibroids was considered. Whole abdominal computed tomography (CT) showed that the left middle and upper abdomen were partially empty, the ileal wall was thickened and swollen, and the thickest part was approximately 2.0 cm. Small bubble density shadows were seen in a part of the intestinal wall, and the corresponding bowel enhancement was not obvious on enhanced scanning. The main portal vein and its branches and the superior mesenteric vein and its branches could be seen with filling defects in the lumen. A small amount of fluid accumulation was seen around the liver, spleen, and intestine. This was considered congestive enteropathy with the portal vein and superior mesenteric vein thrombosis. The uterine shadow enlarged, its inner density was uneven, multiple lumpy and patchy areas of reduced density could be seen, and the enhancement scan was uneven. The presence of multiple uterine fibroids was considered. Many effusion shadows were observed in the pelvic cavity, and no obvious enlarged lymph nodes were found in the retroperitoneum and pelvic cavity. A few patchy cord shadows were seen in the ingested double lower lungs. The boundary was not clear (Figures 1A–F). An upright abdominal radiograph showed a slightly dilated bowel in the abdomen (Figure 1G). Since multiple uterine fibroids caused abnormal vaginal bleeding, DSA uterine angiography showed that the left and right uterine arteries were thickened and disordered, with an enlarged uterus in the parenchyma stage. Repeated angiography after embolization revealed that most of the left and right uterine arteries were blocked (Figures 1H,J). Low-molecular-weight heparin and warfarin were administered for 7 days postoperatively, and the international normalized ratio was maintained at 2.0–3.0. A repeat enhanced CT showed that the main portal vein, the right posterior branch of the liver, the main mesenteric trunk, and some branches were thrombosed and slightly absorbed compared with the previous ones. The proximal branch of the mesentery was painful. In the mid-abdominal region, the wall of the small intestine was thickened and swollen, with surrounding mesentery exudation—multiple uterine fibroids with bleeding and gas. The patient was discharged from the hospital with relief from abdominal pain.

**Figure 2:**
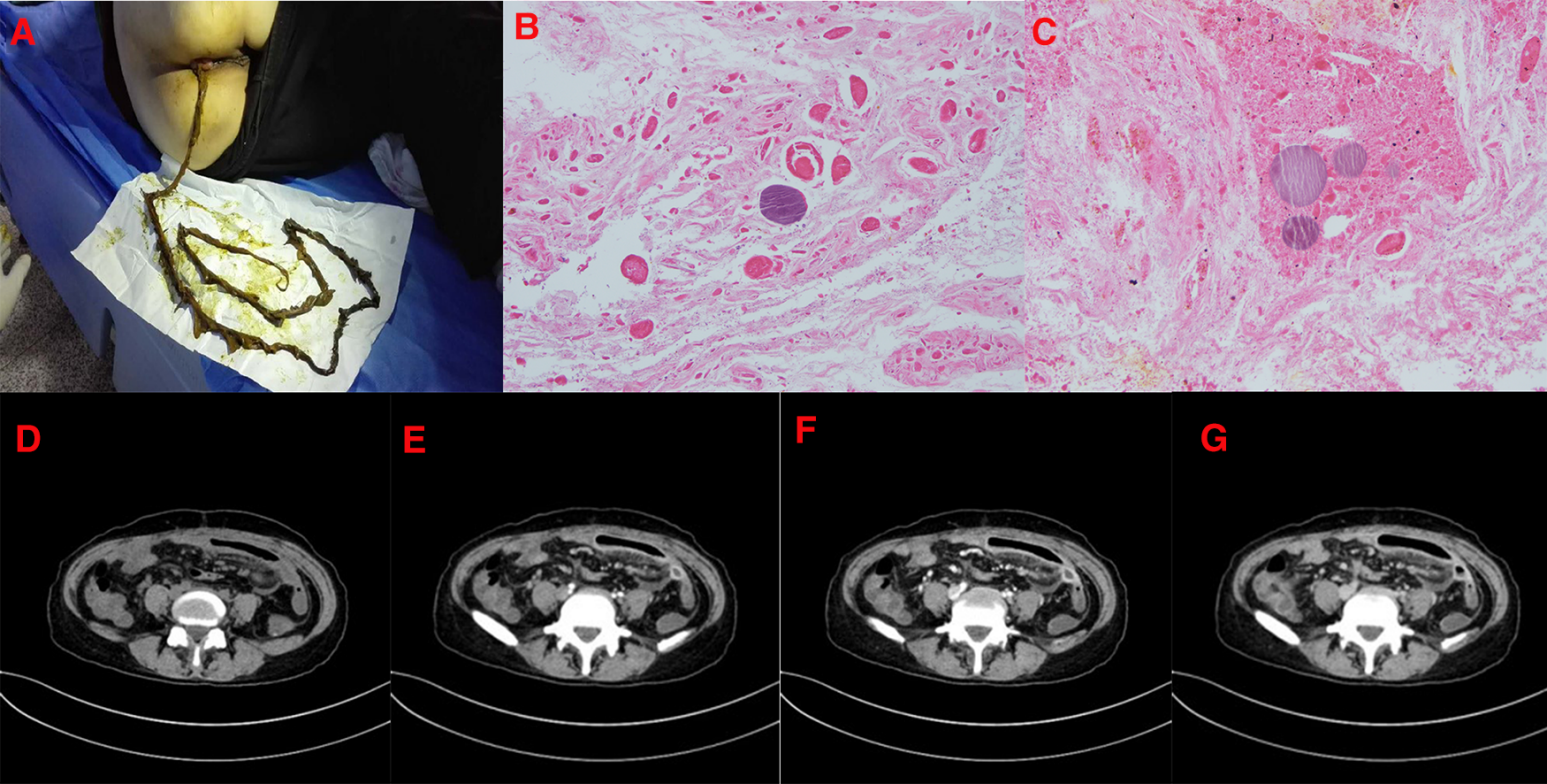
(January 13, 2017) The patient's second visit. (**A**): The shape of the discharge is strip brown. (**B,C**): Under the pathological microscope of the excluded objects, there are large degenerated dead objects, red stained muscle bundle like structures, and no nucleus is found. (**D–G**): Whole abdominal CT after 1 month shows eccentric filling defects in the main portal vein, superior mesenteric vein, and some branch lumens. The extent is significantly smaller than before. The original right posterior thrombus is basically absorbed, and some proximal mesenteric branch veins are unobstructed, which is improved compared with the previous one. In the mid-abdominal region, the segmental small bowel wall is still thickened and swollen, which is reduced compared with the previous one, and now the enhancement is obvious.

One month later, the patient was readmitted with occasional dull pain in the left lower quadrant and a long brown discharge (Figure 2A). An electronic colonoscopy revealed avascular necrosis and tissue exfoliation of the intestinal mucosa. Blood work showed WBC 4.5 × 10^9^/L, N 67.6%, Hb 88 g/L, and Plt 384 × 10^9^/L, coagulation function showed PT 45.8 s, APTT 43.1, INR 4.45, FDP2 5.4 ug/ml, and D-dimer 1.35 mg/L. A whole abdominal CT showed that, while compared with the portal venous phase, eccentric filling defects were seen in the main portal vein, the superior mesenteric vein, and some branch lumens, and the range was significantly smaller than before. The original right posterior thrombus was absorbed, and some proximal mesenteric branch veins were unobstructed, which showed improvement compared with the previous thrombus. In the mid-abdominal region, the segmental small bowel wall was still thickened and swollen, which was reduced compared with the previous one, and the enhancement was obvious. Scattered exudation and a small amount of fluid in the pelvic and abdominal cavities were absorbed earlier (Figures 2D–G). Digital gastrointestinal angiography was used to observe the small intestine of each group over time; the formation of a thick, cord-shaped suspicious fistula in the jejunum of the second group was observed at 4 min, with a range of approximately 10.7 × 1.8 cm. The inner end was inclined to the level of the left vertebral body of T5, and the outer edge was smooth. It was in the fixed state of the left middle and lower abdomen, and its lower edge communicated with the proximal jejunum at the lower end. The lower part of the mucosal folds of the small intestine in Group 4 was limited and slightly expanded in a spring-like fashion. Under the pathological microscope of the discharge, a large area of degeneration and necrosis was observed, a red-stained muscle bundle–like structure was observed, and no nuclei were observed (Figures 2B, c). The patient’s abdominal pain resolved after continuation of warfarin therapy.

**Figure 3:**
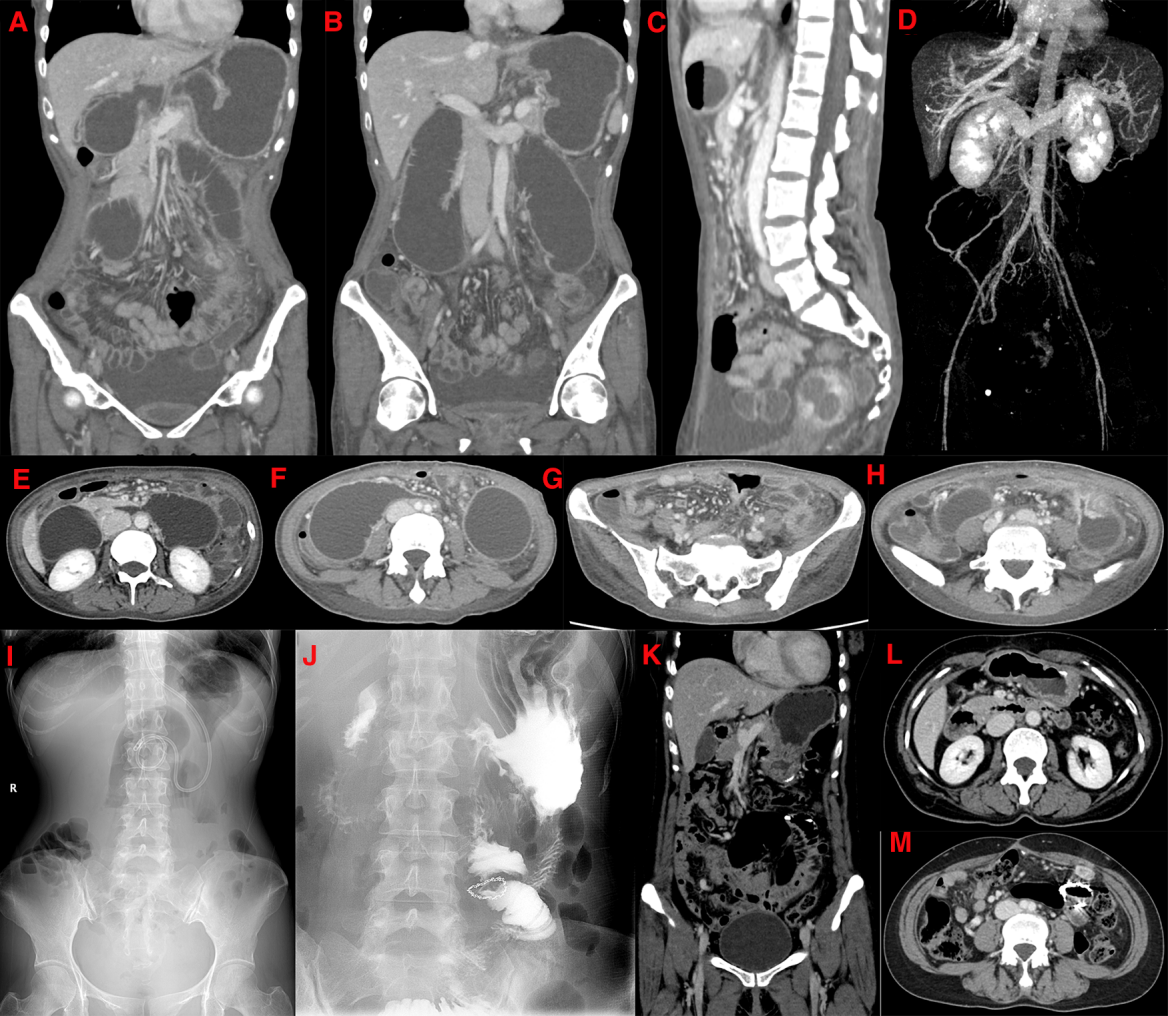
(May 22, 2017) CT examination of the patient's third admission. The coronal view shows a filling defect in the lumen at the origin of the linear filling defect in the superior mesenteric vein (**A,B**). The sagittal view shows a reduced angle between the superior mesenteric artery and the abdominal aorta (**C**). Maximal intensity projection shows a localized widening of the superior mesenteric vein with a hypodense filling defect (**D**). The axial view shows the filling defect sign of the superior mesenteric vein, a marked dilation of the horizontal segment of the duodenum and proximal jejunum, and a local constriction of the intestinal lumen near the origin of the superior mesenteric artery (**E**). The horizontal section of the duodenum is markedly expanded in the transverse axis, with a beak-like narrowing in the near midline area, and the proximal end of the ascending colon is also significantly expanded (**F**). The transverse axis shows inflammatory thickening and edema of the small intestinal wall at multiple sites, with a little mesentery, exudation and local thickening of blood vessels (**G**). Local small bowel dilatation in the left lower abdomen, with local wall thickening and enhancement, and the corresponding organ cavity is narrowing (**H**). (**I**): Preoperative upright abdominal plain film shows multiple lesions in the middle, and lower abdomen gas and liquid levels are seen, and the elastic ring sign is seen in a part of the dilated bowel. (**J**): Postoperative re-examination of the upright abdominal plain film shows changes after a part of the small bowel was resected. A CT scan after resection shows postoperative changes in the small bowel in the coronal view, and no signs of intestinal obstruction are found (**K**). After treatment, there is a little punctate filling defect sign in the superior mesenteric vein, which is more absorbed than before (**L,M**).

**Figure 4:**
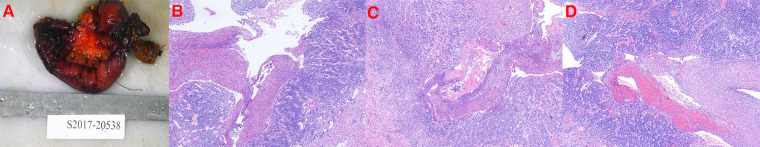
Postoperative pathological findings of the third admission procedure. (**A**): Small bowel removed for partial jejunectomy. (**B–D**): The pathology of the small intestine resected after the operation shows a chronic inflammation of the jejunal mucosa with ulceration, necrosis, and exfoliation of a part of the mucosa, pus on the surface of the intestinal mucosa, and infiltration of many lymphocytes, plasma cells, and a small number of neutrophils and eosinophils in the full thickness of the intestinal wall. Full-thickness blood vessels in the intestinal wall are proliferated, dilated, and congested with hemorrhage, and fibrinous necrosis is seen in some blood vessels.

After 5 months, the patient developed persistent abdominal pain again, aggravated after eating, accompanied by nausea, vomiting, and difficulty in defecation. It was necessary to use a “glycerol enema” to assist in expelling 4–5 times/day. A physical examination revealed a blood pressure of 75/49 mm Hg. Digital total gastrointestinal angiography showed that the descending and transverse parts of the duodenum were dilated, and antiperistalsis could be seen. The small intestines of each group were observed at different times, equivalent to the formation of a cord-shaped suspicious fistula at the proximal end of the jejunum in the second group. The jejunum above it was dilated, and the contrast agent passed through intermittently. Considering the duodenal stasis sign, it was equivalent to the second fistula that could have formed in the proximal small intestine of the group. A whole abdominal CT showed a filling defect in the lumen at the origin of the superior mesenteric vein, which was considered a thrombosis at the origin of the superior mesenteric vein. After myomectomy and bilateral uterine artery embolization, lump-like shadows with abnormal density were seen on the posterior wall of the uterus, and the enhancement showed uneven and obvious enhancement, with a size of about 5.2 × 3.9 × 4.3 cm (Figures 3A–C, E–H). The maximal intensity projection (MIP) image showed that the local lumen of the superior mesenteric vein was slightly widened, and a longitudinal linear hypodense filling defect was observed (Figure 3D). The upright abdominal radiograph showed multiple gas-liquid levels in the middle and lower abdomen, and the elastic ring sign was seen in a part of the dilated bowel (Figure 3I). The current diagnosis was incomplete intestinal obstruction with the portal vein and superior mesenteric vein thromboses. Therefore, an exploratory laparotomy was performed, and partial jejunal resection and gastrointestinal bypass anastomosis were performed during the operation (Figure 4A). A postoperative pathological examination showed a chronic inflammation of the jejunal mucosa with ulceration, partial mucosal necrosis and exfoliation, pus on the surface of the intestinal mucosa, infiltration of many lymphocytes and plasma cells, and a small number of neutrophils and eosinophils in the full thickness of the intestinal wall. Vascular hyperplasia, dilation and congestion with hemorrhage, and fibrinoid necrosis were observed in some blood vessels (Figure 4B–D). There were 26 lymph nodes around the jejunum, indicating reactive hyperplasia. A postoperative re-examination CT showed some changes after small bowel resection. The anastomotic stoma was unobstructed, multiple gases and fluid levels in the original abdomen had disappeared before the operation, and no signs of intestinal obstruction were found. A small punctate filling defect in the superior mesenteric vein was absorbed earlier (Figure 3K–M). The upright abdominal radiograph showed that the multiple gas–liquid levels in the original abdomen had disappeared before surgery (Figure 3J). The abdominal pain disappeared after the operation, warfarin was continued after discharge, and the symptoms have not recurred since.

## Discussion

This is the first reported case of intact necrotic intestinal mucosal tissue shedding and necrosis due to SMV thrombosis. The clinical manifestations are unique. The history of thrombosis and imaging examinations suggested SMV thrombosis. The main clinical feature of patients with SMV thrombosis is persistent abdominal pain lasting 7–10 days. Gastrointestinal symptoms also manifest as abdominal distension, which may accompany bleeding (6). These undirected symptoms can affect the diagnosis of SMV thrombosis, lead to untimely treatment, and eventually result in ischemic intestinal necrosis. Therefore, for unexplained ascites, splenomegaly, abdominal pain, abdominal distension, peritonitis, portal hypertension, and other symptoms, an abdominal imaging examination should be performed to exclude SMV thrombosis and avoid delayed diagnosis (7). Appendicitis symptoms with right lower quadrant tenderness may occur in some SMV thrombosis (8).

The diagnosis of SMV thrombosis mainly relies on imaging and color Doppler ultrasonography, which is simple and low in radiation, and so can be the first choice for the preliminary examination. If an abnormality is found, it can be confirmed using further enhanced CT scans. Enhanced CT is an important diagnostic method for SMV thrombosis with an accuracy rate of up to 90% (9). In addition, laboratory tests for SMVT are neither sensitive nor specific (10), and elevated plasma D-dimer levels may indicate an early diagnosis of SMVT (11). When SMV thrombosis is unclear, systemic heparin anticoagulation and urokinase thrombolysis are administered as the initial therapy. Studies have shown that early anticoagulation therapy can effectively reduce complications and thrombus enlargement without intestinal ischemia or necrosis (9). When SMV thrombosis is confirmed, interventional or surgical methods can be used for performing thrombectomy. In recent years, the application of interventional therapy for this type of vascular disease has become increasingly extensive, which can significantly shorten hospitalization time and trauma, accelerate postoperative recovery, and restore the blood vessels of the infarcted intestinal tract as soon as possible (2). The surgical methods of interventional therapy include fragmentation, crushing, and suction, especially percutaneous mechanical thrombectomy in the treatment of thrombus, which can reduce the dose of thrombolytic agents and speed up the postoperative recovery of patients (12, 13). In 46 patients with portal vein PV-SMV thrombosis, Liu et al. found that the efficacy of direct injection of a thrombolytic agent into the catheter significantly improved the thrombolytic effect and reduced related bleeding complications (14). In intestinal ischemic diseases, the incidence of postoperative acute renal failure, pulmonary failure, and death in patients receiving interventional therapy is significantly reduced compared with traditional surgical treatment, which can significantly improve the prognosis of patients (15). However, if strangulated ileus or bowel perforation is life-threatening, immediate abdominal exploration is required to remove the necrotic bowel (16). Our patient did not develop shock or life-threatening conditions in the early stages and was therefore managed by conservative treatment. Four months later, the patient presented with a worsening abdominal pain and decreased blood pressure. This suggested that a surgical resection of the necrotic bowel is unavoidable when anticoagulant therapy is ineffective or there are signs of peritonitis or manifestations of bowel avascular necrosis (17).

The patient had recurrent thrombosis, but the specific etiology was unclear, which may be related to genetic factors. For example, mutations in PROS1 lead to protein S deficiency, and MTHFR or Factor V Leiden mutations can lead to hereditary thrombosis (18). Acquired viral infections or drug factors can also cause thrombosis. Therefore, for patients with recurrent thrombosis, it is necessary to investigate their past medical history and drug use history. If necessary, genetic testing and family medical history research can be done to determine a familial hereditary thrombophilia. For family members with hereditary factors, if there is a pathogenic gene mutation, anticoagulants can be used in advance to prevent thrombosis and reduce the risk of thrombosis.

In our case, the patient can use non-vitamin-K-dependent oral anticoagulants (NOAC) such as drug-targeting factors Xa (rivalsaban, apixaban) or thrombin (dabigatran) (19) to prevent recurrent thrombosis; a new oral anticoagulant can also be used to prevent recurrent thrombosis, or genetic testing can be performed to exclude the possibility of genetic causes. Therefore, we can further investigate the genetic background of the patient’s family to make an accurate and effective diagnosis and provide treatment. Early preventive measures can also be provided for family members who may have a thrombotic event. By reporting the characteristics of this case, the clinical manifestations of SMV thrombosis were enriched, and a new basis for the diagnosis of SMV thrombosis was provided.

## Data Availability

The original contributions presented in the study are included in the article/Supplementary Material, and further inquiries can be directed to the corresponding authors.
